# P-1246. Effect of BV100 on the Pharmacokinetics of Midazolam and its metabolite 1-hydroxymidazolam in healthy volunteers

**DOI:** 10.1093/ofid/ofae631.1428

**Published:** 2025-01-29

**Authors:** Pierre Delique, Lisa Husband, Christian Kemmer, Geza Lakner, Glenn Dale

**Affiliations:** BioVersys SAS, Lille, Ile-de-France, France; BioVersys SAS, Lille, Ile-de-France, France; BioVersys AG, Basel, Basel-Stadt, Switzerland; CRU Hungary Kft, Kistarcsa, Budapest, Hungary; BioVersys AG, Basel, Basel-Stadt, Switzerland

## Abstract

**Background:**

We identified rifabutin as having potent antibacterial activity towards *Acinetobacter baumannii* under iron limiting conditions. BV100 (rifabutin for infusion) is under development for the treatment of serious infections due to *A. baumannii*

A single center, open-label, 3-period fixed-sequence, phase I clinical drug-drug interaction study was conducted to evaluate the effect of BV100 on the pharmacokinetics (PK) of midazolam and its metabolite 1-hydroxymidazolam on healthy subjects.

**Methods:**

The study included nonchildbearing females and males aged 18 to 55. The study was divided in three periods : On Day 1 (Period 1), each participant received a single 5 mg oral dose of midazolam syrup. After a 24-hour washout on Day 2, BV100 was given as a 300 mg intravenous infusion over 2 hours every 12 hours for 8 doses from Day 3 to Day 6 (Period 2). On Day 7 (Period 3), the 9th dose of BV100 was administered alongside a single 5 mg oral dose of midazolam. PK parameters for midazolam and its metabolite were assessed in plasma samples on Day 1 after midazolam administration alone and on Day 7 after midazolam administration following 8 BV100 doses from Day 3 to Day 6.

**Results:**

No deaths, no SAEs, or other significant AEs occurred during this study. All 23 study subjects were eligible for safety analysis. Sixteen out of 23 subjects completed all 3 periods of the study. No clinically relevant effects of BV100 on clinical laboratory profiles (haematology, biochemistry, haemostasias, urinalysis) or vital signs (blood pressure, heart rate, respiration rate, body temperature) were observed in this study.

Midazolam and 1-OH-midazolam plasma concentrations were higher when administered alone on Day 1 (Cmax = 33.36 ng/mL; AUC_0-tlast_ = 99.20 h*ng/mL) compared to co-administration with BV100 on Day 7 (Cmax = 25.43 ng/mL; AUC_0-tlast_ = 50.81 h*ng/mL). The elimination parameters indicate faster elimination for both midazolam and its metabolite on Day 7 compared to Day 1.

**Conclusion:**

BV100 is considered a mild and weak inducer of midazolam according to EMA and FDA criteria, respectively. Multiple 300 mg IV doses of BV100, administered with and without midazolam to healthy subjects, were safe and well tolerated.

**Disclosures:**

**All Authors**: No reported disclosures

